# Ghrelin Does not Alter Aortic Intima-Media Thickness and Adipose Tissue Characteristics in Control and Obese Mice

**Published:** 2013-08

**Authors:** Zoya Tahergorabi, Bahman Rashidi, Majid Khazaei

**Affiliations:** 1 Department of Physiology, Isfahan University of Medical Sciences, Isfahan, Iran; 2 Department of Anatomy, Isfahan University of Medical Sciences, Isfahan, Iran

**Keywords:** Adipocyte, Atherosclerosis, Ghrelin, Obesity

## Abstract

***Objective(s):*** Atherosclerosis is a chronic immune-inflammatory disease that generally leads to ischemic heart disease. Ghrelin has several modulatory effects on cardiovascular system. In this study, we investigated the effect of ghrelin on aortic intima-media thickness, size and the number of adipocyte cells in obese and control mice.

***Materials and Methods:*** This study was conducted on 24 male C57BL/6 mice. The animals were divided into four groups: control, obese (received high fat diet), control+ghrelin (injected with 100 µg/Kg subcutaneously, bid) and obese+ghrelin (n=6 each). After 10 days, animals were sacrificed and epididymal adipose tissue and thoracic aortae were removed. Adipocyte cell number, size and aortic intima-media thickness were evaluated.

***Results:*** Ghrelin did not change adipocyte cell number and size and aortic intima-media thickness in obese and control mice. In this study, high fat diet significantly decreased the number of adipocyte cells while increased their size (*P*<0.05). Ghrelin administration had no significant effect on adipocyte cell number and size in obese and control groups (*P* >0.05). In addition, it could not alter aortic intima-media thickness in both groups.

***Conclusion:*** Although ghrelin has several cardiovascular effects, it seems that it could not alter the size and number of adipocyte cells and aortic intima-media thickness in diet-induced obese mice.

## Introduction

Atherosclerosis is known as a chronic immune-inflammatory disease (1) generally leading to ischemic heart disease (IHD). IHD is the leading cause of death in the developed countries as well as worldwide (2). Atherosclerosis is characterized by endothelial dysfunction, lipoprotein oxidation, leukocyte infiltration and accumulation of cholesterol deposits in macrophages in large and medium sized arteries. The earliest histological manifestation of arterial lesion is fatty streak formation. The fatty streak leads to atherosclerotic plaque, acute and chronic luminal obstruction and limits the supply of blood and oxygen to the target organs (3-4). 

Obesity that is defined as excessive body fat (5) is considered as an independent risk factor for coronary heart disease(CHD), hypertension and diabetes mellitus (6). Obesity is a major public health concern due to increasing at an alarming rate especially in developed countries. Based on the statistics, the number of obese people all over the world, in 2005 has been recorded as 396 million cases which is predicted to increase to 573 million in 2030 (7). Obesity is considered a low grade inflammation state and in the pathophysiology of atherosclerosis also inflammation plays a crucial role (8). Furthermore, adipocyte as well as macrophages may uptake and degrade oxidized low-density lipoprotein (LDL). Dysfunction of adipocytes in obesity can lead to impaired uptake and degradation of oxidized LDL and promotion of atherosclerosis (9). 

Ghrelin, a 28 amino acid peptide hormone, is an endogenous ligand for the growth hormone secretagogue receptor (GHS-R)(10). Ghrelin is largely secreted by the X/A-like cells in the oxyntic mucosa of the stomach but smaller amount is produced by other organs such as heart, lung, kidney, pancreas, gonads, thyroid, adrenal, pituitary and hypothalamus (11). In addition to growth hormone (GH) secretion, it stimulates food intake and regulates appetite, body weight and metabolism of glucose and fat (12). 

**Figure 1:**
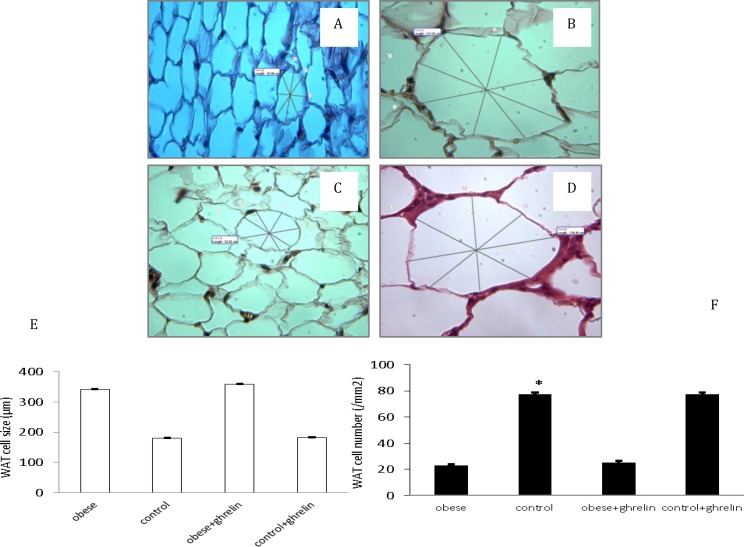
Effect of high-fat diet and ghrelin administration on white adipose tissue (WAT). The histological sections were stained with hematoxylin& eosin. A: control; B: obese; C: control+ghrelin; D: obese+ghrelin. HFD loading significantly increased the size of epididymal adipocyte cells and decreased the cell number. Ghrelin administration did not change adipocyte cell size and number of epididymal adipocyte cells (E&F). Data is shown as mean ± SEM (n=6). * *P*<0.05 as compared to the control group

The potential regulating mechanism of ghrelin on atherosclerosis is not clear. Since, endothelial dysfunction, inflammation and oxidative stress are involved in the pathophysiology of atherosclerosis, some studies have demonstrated that ghrelin may be involved in atherosclerosis processes, although the results are contradictory (13-15). In this study, we investigated the effect of ghrelin on adipocyte cell size and number and aortic intima-media thickness in normal and diet-induced obese mice. 

## Materials and Methods

***Animals***

 A total of 24 male mice C57BL/6J, weighing 20-30 g, and 5 weeks old were purchased from Pasteur Institute of Tehran, Iran. They were housed on a 12 hr light-dark cycle at 25^°^C room temperature with free access to food and water *ad libitum*. The ethical committee of Isfahan University of Medical Sciences approved the study protocol. The animals were divided into four groups: obese, control, obese+ghrelin and control+ghrelin (n=6). 

***Animals diet and their treatment***

 For induction of diet-induced obesity, the obese group was fed a commercial high fat diet (HFD; BioServ Co., Cat #F3282, USA) for 14 weeks (16), while, the control groups were fed the standard mouse chow. All animals had free access to food and water during the study. Body weight of animals was monitored on a weekly basis. 

 After 14 weeks, half of the obese and control animals received ghrelin. Ghrelin was obtained from Tocris Co. (Bristol, UK) and 100 µg/Kg was injected subcutaneously, bid (17). After 10 days, animals were sacrified and epididymal adipose tissues and thoracic aortae were removed.

***Histological examination***

Adipose tissues and thoracic aortae were removed and fixed in 10% formalin. Then, they were dehydrated and embedded in paraffin. Tissue blocks were sectioned into 5 µm thickness and stained with hematoxylin and eosin (H&E). Adipocyte cell numbers were counted in 5 different fields through the camera of light microscope equipped with computerized image analysis software (advanced Motic image 3.2). For determination of cell size, diameter of adipocyte cells in 10 cells for each specimen was analyzed and recorded. Aortic intima-media thickness was measured from the endothelial surface to the adventitia in 13 different fields of the samples of each animal (18).

***Statistical analysis***

Data were subjected to one-way analysis of variance (ANOVA) with multiple comparison test using LSD. A

significant difference was determined at 0.05 probability Level. All statistical analyses of data were performed using SPSS (version 16). The data are reported as mean values ± SEM. 

## Results

***Effect of ghrelin on adipocyte cell number***

Adipocyte cell number was significantly different between obese and control groups (23.1±0.95 vs. 77.7±11.56 number/field, respectively; *P*<0.05). Ghrelin administration did not affect adipocyte cell number in obese (25.4±2.76 vs. 23.1±0.95 number/field; *P*>0.05) and control groups (77.6±4.87 vs. 77.7±11.56 number/field;* P* >0.05) (Figure 1 A-E).

***Effect of ghrelin on adipocyte cell size***

Adipocyte cell size in white adipose tissues in obese animals was significantly higher than control group (341.27±24.02 vs. 179.52±5.7 μm, respectively; *P*<0.05). Ghrelin could not alter adipocyte cell size in obese (358.74±25.48 vs. 341.27±24.02 μm;* P*>0.05) and control groups (182.28±10.3 vs. 179.52±5.7 μm;* P* >0.05) (Figure 1 A-D&F).

***Effect of ghrelin on aortic Intima-Media thickness***

**Figure 2:**
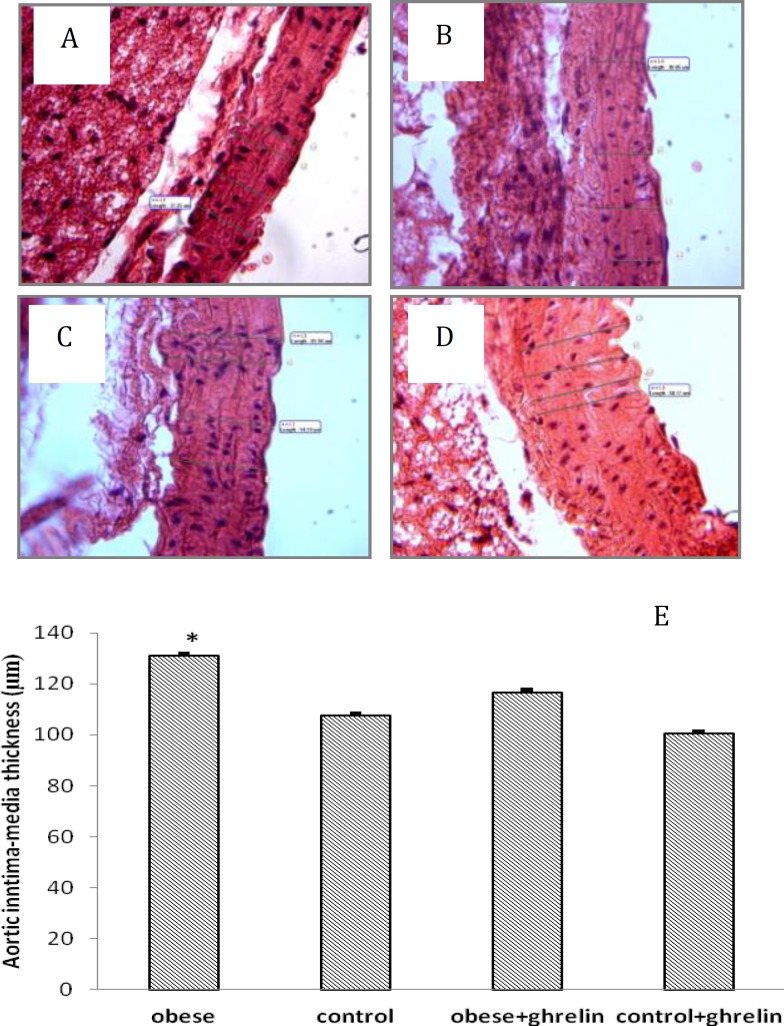
Effects of high-fat diet loading and ghrelin administration on aortic intima-media thickness. The histological sections of aorta were stained with hematoxylin& eosin (A-D); A: control; B: obese; C: control+ghrelin; D: obese+ghrelin. HFD loading significantly increased aorticintima-media thickness in obese compared to control. Ghrelin administration did not alter aortic intima-media thickness in obese and control group (E). Aortic intima-media thickness as the mean value from the endothelial surface to the adventitia was recorded from 13 different locations spanning the entire cross-section. Data is shown as mean ± SEM (n=6). * *P*< 0.05 as compared to the control group

The aortic rings from control and obese mice stained by H&E stains are shown in Figure 2 (A-D). Aortic intima-media thickness in obese animals was significantly higher than control group (130.86± 8.77 vs. 107.51±6.71 μm, respectively; *P*<0.05). Administration of ghrelin did not affect aortic intima-media thickness in obese (116.68± 6.78 vs. 130.86±8.77 μm; *P*>0.05) and control groups (100.45±6.36 vs.107.51±6.71 μm; *P*>0.05) (Figure 2-E). 

## Discussion

This study examined the effect of ghrelin on white adipose tissue characteristics including the number and size of adipocyte cells and aortic intima-media thickness in normal and diet-induced obese mice. Our results showed that ghrelin had no significant effect on the number and size of adipocyte cell and aortic intima-media thickness in obese and control mice. 

Epididymal fat in rodents is the representative of visceral fat depot because it has many similarities to the features of visceral fat in humans (19). Several evidences have shown that larger adipocyte cells exhibit increased lipolysis and release more free fatty acid (FFA) into the circulation that can lead to fatty acid toxicity in insulin responsive organs. Thus adipocyte cell size, but not adipocyte cell number can be correlated with insulin resistance (20, 21). Currently, because of the close relationship between obesity and type 2 diabetes, the new term of disabesity is used (22). Therefore, in our study, increased adipocyte cell size in obese mice has created a condition which is similar to type 2 diabetes and insulin resistance.

Obesity is considered as an independent risk factor for atherosclerosis and coronary artery disease(6). The earliest histological manifestation of arterial lesion is fatty streak formation. The fatty streak can lead to atherosclerotic plaque, acute and chronic luminal obstruction and limit the supply of blood and oxygen to the target organs (3,4). As we expected, the intima-media thickness in aortae of obese animals was higher than the control. However, in the present study, administration of ghrelin not only could not alter adipose tissue cell size and number, but also it had no effect on artic intima-media thickness in control and obese animals. 

Ghrelin, a 28 amino acid peptide hormone, is an endogenous ligand responsible for the growth hormone secretagogue receptor (GHS-R). Ghrelin is largely secreted by oxyntic mucosa of the stomach and in addition to leading to GH (growth hormone) secretion, it stimulates food intake and regulates appetite, body weight and metabolism of glucose and fat (11). Several studies reported the effect of ghrelin on cardiovascular system including protective role on endothelium, enhancement of left ventricular function during ischemia-reperfusion injury in rodents (23), improvement of cardiac function and decrement of vascular resistance in chronic heart failure (CHF) in humans (24). In the present study, it was shown that ghrelin had no significant effect on the number and size of adipocyte cell. Davies *et al*, showed that intravenous infusion of acylated ghrelin, unacylated ghrelin or ghrelin specific ligand (L-163, 255) increases the white adipose tissue (WAT) mass in retroperitoneal and perirenal without having any significant influence on inguinal or epididymal WAT weight and any of the parameters of adiposity (25). In addition, other studies demonstrated that ghrelin receptor expression was not increased in obese mice with HFD in comparison with regular diet-13 month old mice (26) which are consistent with our results. Furthermore, there are conflicting reports regarding the effects of ghrelin on atherosclerosis process. Some studies reported a relation between plasma ghrelin level and the severity of atherosclerosis (27). Although, the potential mechanism of ghrelin on atherosclerosis is not clear, it is indicated that ghrelin receptors are up-regulated in atherosclerotic arteries (14). Furthermore, ghrelin may reduce atherosclerosis through its anti-oxidative and anti-inflammatory effects (28). However, in some pathophysiological conditions such as type II diabetes, ghrelin may increase atherosclerosis risk through increase adhesive molecules (29). In addition, it is demonstrated that in kidney transplant patients, ghrelin does not have protective effect on atherosclerosis (30). In agreement with our results, a recent study indicated that LDL receptor deficient mice C57BL/6 as a mouse model of atherosclerosis fed with saturated fat diet and double knock-out mice (GHSr/ LDL^-/-^) showed remarkable peak in very low density lipoprotein (VLDL), low density lipoprotein ( LDL ) cholesterol (15). Furthermore, GHSr/ LDL^-/- ^mice exhibited notable atherosclerosis in the aortic arch and aorta fatty streak formation which was not different between gherlin-receiving and non-gherlin-receiving group. 

## Conclusion

In conclusion, although ghrelin has several cardiovascular effects, it seems that it has no effect on aortic intima-media thickness and size and the number of adipocyte cells in diet-induced obese mice. Of course, the exact role of ghrelin on atherogenesis needs further investigations.
